# The clinical use of circulating microRNAs as non-invasive diagnostic biomarkers for lung cancers

**DOI:** 10.18632/oncotarget.21644

**Published:** 2017-10-04

**Authors:** Yanlong Yang, Zaoxiu Hu, Yongchun Zhou, Guangqiang Zhao, Yujie Lei, Guangjian Li, Shuai Chen, Kai Chen, Zhenghai Shen, Xiao Chen, Peilin Dai, Yunchao Huang

**Affiliations:** ^1^ Department of Thoracic Surgery I, The Third Affiliated Hospital of Kunming Medical University (Yunnan Cancer Hospital, Yunnan Cancer Center), Kunming 650118, PR China; ^2^ Department of Pathology, The Third Affiliated Hospital of Kunming Medical University (Yunnan Cancer Hospital, Yunnan Cancer Center), Kunming 650118, PR China; ^3^ Cancer Research Institute of Yunnan Province, The Third Affiliated Hospital of Kunming Medical University (Yunnan Cancer Hospital, Yunnan Cancer Center), Kunming 650118, PR China; ^4^ Key Laboratory of Lung Cancer Research of Yunnan Province, The Third Affiliated Hospital of Kunming Medical University (Yunnan Cancer Hospital, Yunnan Cancer Center), Kunming 650118, PR China; ^5^ International Joint Laboratory of High Altitude Regional Cancer of Yunnan Province, The Third Affiliated Hospital of Kunming Medical University(Yunnan Cancer Hospital, Yunnan Cancer Center), Kunming 650118, PR China; ^6^ Department of Radiation Oncology, The Third Affiliated Hospital of Kunming Medical University (Yunnan Cancer Hospital, Yunnan Cancer Center), Kunming 650118, PR China

**Keywords:** lung cancer, circulating microRNAs, diagnostic value, meta-analysis

## Abstract

Many studies have investigated the diagnostic role of circulating microRNAs (miRNAs) in patients with lung cancer; however, the results still remain inconclusive. An updated system review and meta-analysis was necessary to give a comprehensive evaluation of diagnostic role of circulating miRNAs in lung cancer. Eligible studies were searched in electronical databases. The sensitivity and specificity were used to plot the summary receiver operator characteristic (SROC) curve and calculate the area under the curve (AUC). The between-study heterogeneity was evaluated by Q test and I^2^ statistics. Subgroup analyses and meta-regression were further performed to explore the potential sources of heterogeneity. A total of 134 studies from 65 articles (6,919 patients with lung cancer and 7,064 controls) were included for analysis. Overall analysis showed that circulating miRNAs had a good diagnostic performance in lung cancers, with a sensitivity of 0.83, a specificity of 0.84, and an AUC of 0.90. Subgroup analysis suggested that combined miRNAs and Caucasian populations may yield relatively higher diagnostic performance. In addition, we found serum might serve as an ideal material to detecting miRNA as good diagnostic performance. We also found the diagnostic role of miRNAs in early stage lung cancer was still relatively high (the sensitivity, specificity and an AUC of stage I/II was 0.81, 0.82 and 0.88; and for stage I, it was 0.80, 0.81, and 0.88). We also identified a panel of miRNAs such as miR-21-5p, miR-223-3p, miR-155-5p and miR-126-3p might serve as potential biomarkers for lung cancer. As a result, circulating miRNAs, particularly the combination of multiple miRNAs, may serve as promising biomarkers for the diagnosis of lung cancer.

## INTRODUCTION

Currently, the mortality rate of lung cancer is still the highest among all cancers in both men and women [1]. The data indicated that an estimated 733,300 new lung cancer cases and 610,200 lung cancer deaths would occur in China in 2015 [2]. According to National Center for Health Statistics in 2017, there were an estimated 222,500 new cases of lung cancer diagnosed, and 155,870 died from lung cancer in the United States [3]. Lung cancer can be divided into two major forms: non-small cell lung cancer (NSCLC) (about 85% of all lung cancers) and small cell lung cancer (SCLC) (about 15%) [4]. Despite advances in early detection and standard treatment, approximately two thirds of NSCLC cases were diagnosed at locally advanced (27.6%) or metastatic (38.1%) disease as the typically asymptomatic at early stages [5]. Thus, the earlier detection of lung cancer would be great meaningful to improve the prognosis of this lethal disease.

Computed tomography (CT), especially low-dose CT is currently widely used as the screening method for lung cancer. But some concerns may exist, such as high false-positive rates, potential over-diagnosis, excessive cost and radiation risk [6]. Recent years, more attentions have been paid in circulating microRNAs (miRNAs) in the field of biomarker discovery for cancer. MiRNAs are short (typically 18-25 nucleotides), single-stranded and highly conserved non-coding RNAs which could negatively regulate gene expression at post-transcriptional level by binding the 3′-untranslated region of target mRNAs, resulting in either mRNA degradation or translational repression [7]. Passively leaked or actively transported from cells, circulating miRNAs could be stably detected in blood and have been used as biomarkers for diagnosis, prognosis or monitoring curative effect in various cancers including lung cancer [7–9].

The diagnostic role of single and various sets of circulating miRNAs in lung cancer have been investigated by many studies. However, the results from individual studies were inconsistent, and the miRNA signatures identified from individual studies were different from each other. Therefore, we conducted this systematic review and meta-analysis to further evaluate the clinical applicability of circulating miRNAs as biomarkers for the diagnosis of lung cancer.

## RESULTS

### Eligible studies

**Figure 1:**
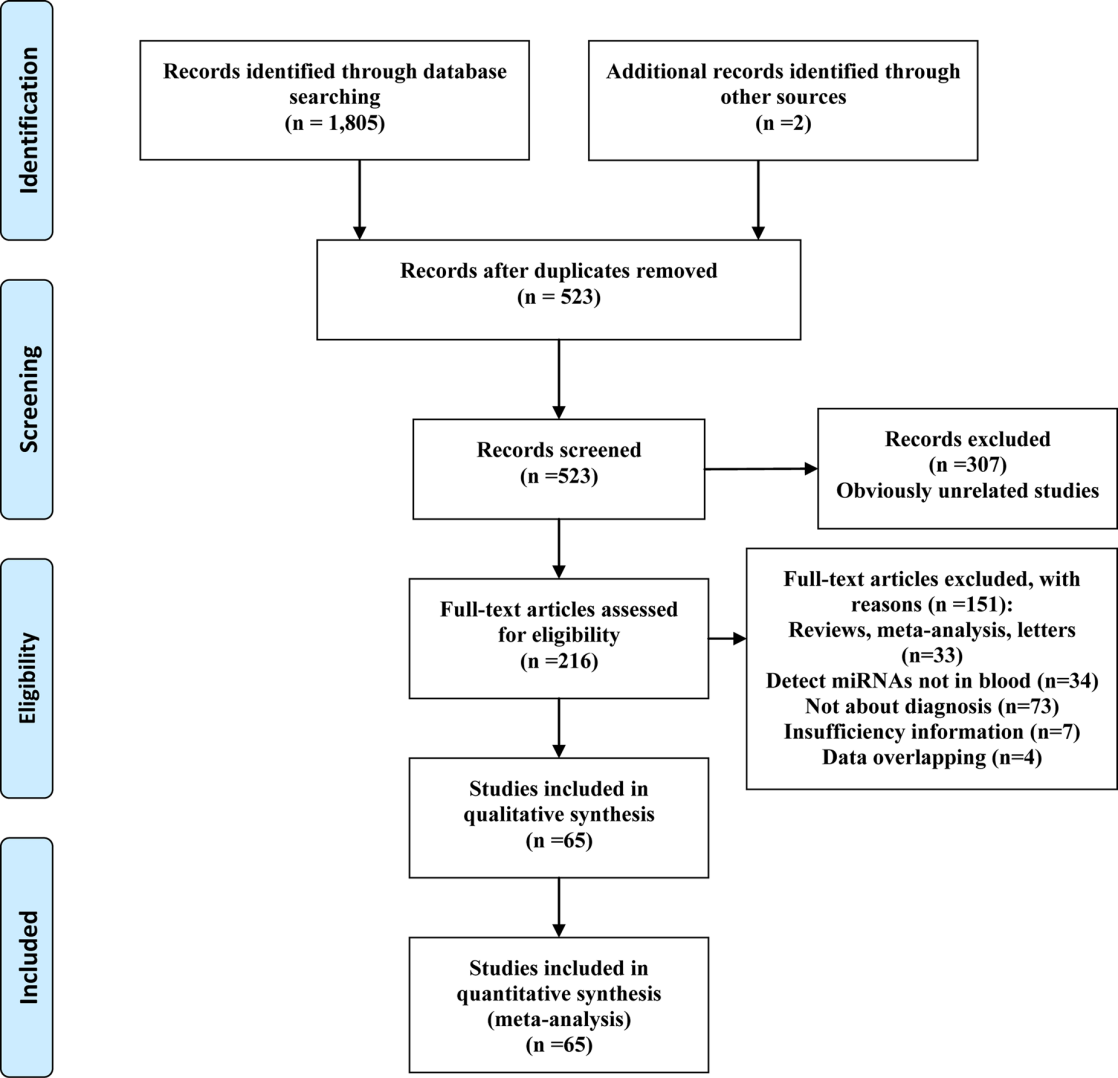
Flow diagram of the selection procedure of studies

The present work followed the guidelines for systematic reviews and meta-analyses (PRISMA) [10]. 523 articles were identified from initial screen. After reviewing the titles and abstracts, 307 articles were excluded because they obviously did not meet our selection criteria. The remaining 216 articles were further checked by screening the full texts. Finally, a total of 134 studies from 65 articles including 13,983 samples (6,919 patients with lung cancer and 7,064 controls) were qualified for our analysis [11–75]. The process of article selection is summarized in Figure 1. The main characteristics of the included articles are listed in Supplementary Table 1. The qualities of the selected studies according to QUADAS-2 guidelines are showed in Supplementary Figure 1.

### Pooled diagnostic accuracy of miRNAs

**Figure 2:**
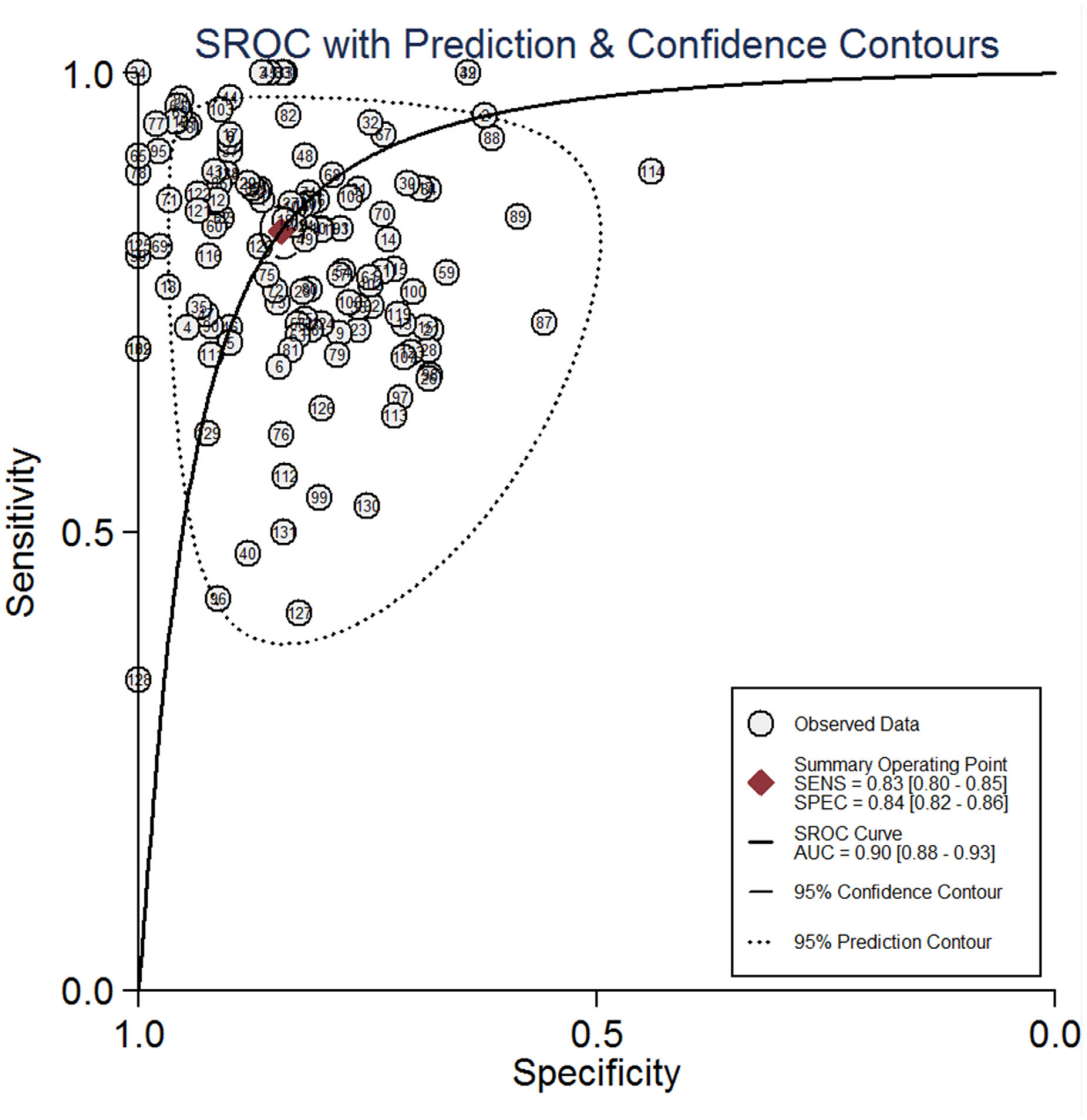
The summary receiver operator characteristic (SROC) curves of circulating miRNAs test for the diagnosis of lung cancer patients in overall population

**Figure 3:**
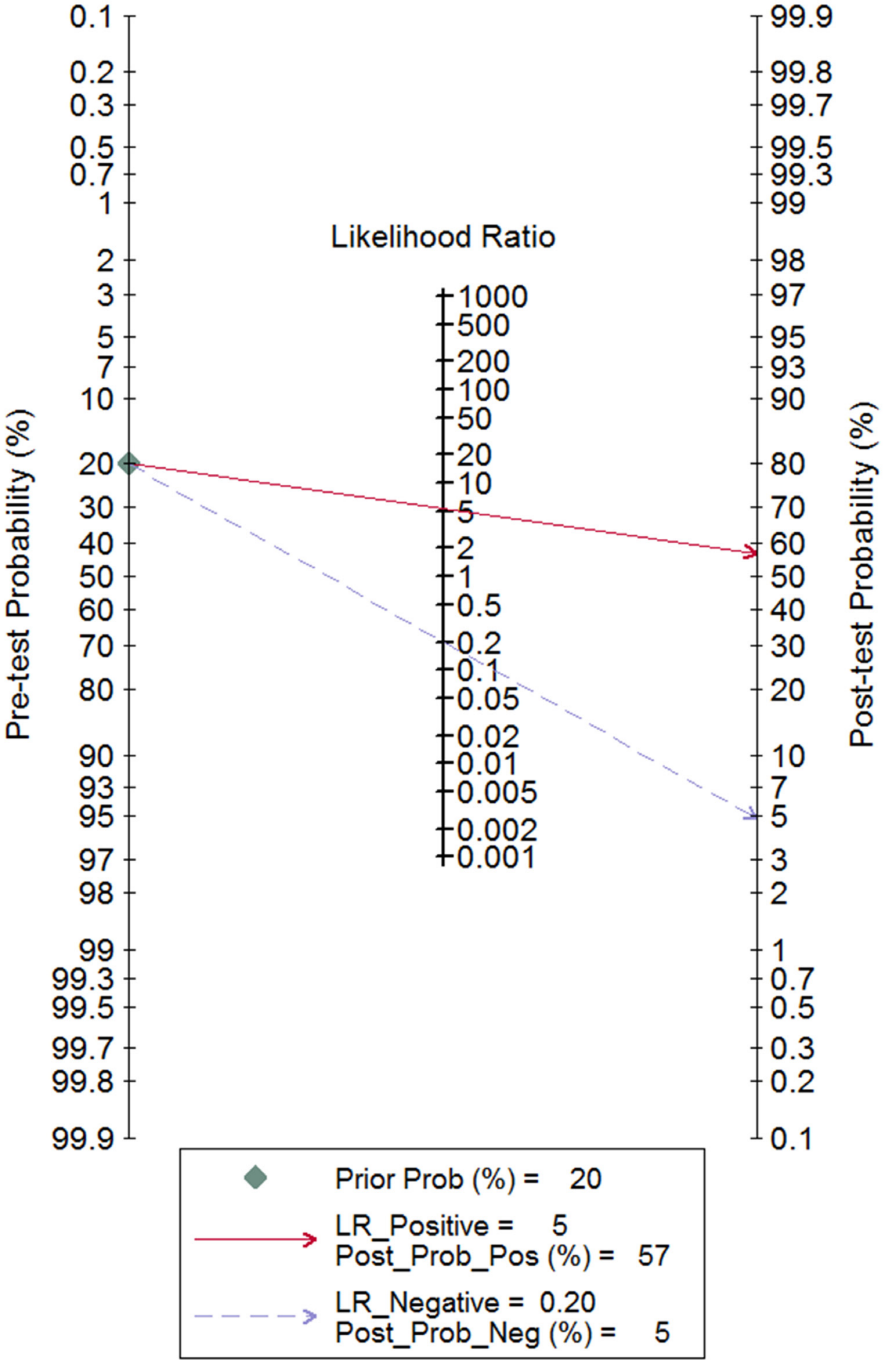
Fagan diagram evaluating the positive likelihood ratio (PLR) and negative likelihood ratio (NLR)

All 134 studies were available for analysis the overall predictive accuracy of miRNAs for detecting lung cancer. The random effects model was applied. The pooled sensitivity was 0.83 (95% CI: 0.80–0.85), the specificity was 0.84 (95% CI: 0.82–0.86), the pooled positive likelihood ratio (PLR) was 5.3(95% CI: 4.7–6.0), the negative likelihood ratio (NLR) was 0.20 (95% CI: 0.18–0.23) and the diagnostic odds ratio (DOR) was 26(95% CI: 21–33) (Table 1). The corresponding summary receiver operator characteristic (SROC) curve is shown in Figure 2. The area under the curve (AUC) value was 0.90 (95% CI: 0.88–0.93) in the overall SROC curve. The Fagan diagram in Figure 3 illustrates the post-test probabilities of circulating miRNAs in lung cancer diagnosis. Our analysis suggested circulating miRNAs may yield a relatively high diagnostic accuracy for detecting lung cancer patients.

**Table 1:** The main results of meta-analysis

Analysis	No. of studies	SEN (95%CI)	SPE (95%CI)	PLR (95%CI)	NLR (95%CI)	DOR (95%CI)	AUC (95%CI)
**Overall**	134	0.83(0.80,0.85),I^2^=89.3%,p<0.001	0.84(0.82,0.86),I^2^=80.58%,p<0.001	5.3(4.7,6.0)	0.20(0.18,0.23)	26(21,33)	0.90(0.88,0.93)
**Subgroup 1: race**							
Asian	86	0.81(0.78,0.84),I^2^=90.67%,p<0.001	0.84(0.82,0.86),I^2^=80.61%,p<0.001	5.1(4.4,5.9)	0.22(0.19,0.26)	23(18,30)	0.90(0.87,0.92)
Caucasian	39	0.88(0.83,0.91),I^2^=84.53%,p<0.001	0.86(0.82,0.89),I^2^=85.38%,p<0.001	6.2(4.7,8.1)	0.14(0.11,0.20)	43(26,68)	0.93(0.91,0.95)
Mixed	9	0.76(0.72,0.79),I^2^=0.0%,p=0.68	0.82(0.79,0.85),I^2^=0.0%,p=0.59	4.3(3.6,5.2)	0.29(0.25,0.34)	15(11,20)	0.86(0.83,0.89)
**Subgroup2: miRNA profiling**							
Single miRNA	98	0.79(0.76,0.82),I^2^=88.46%,p<0.001	0.78(0.76,0.81),I^2^=81.27%,p<0.001	3.7(3.3,4.1)	0.27(0.23,0.31)	14(11,16)	0.85(0.82,0.88)
Multiple miRNA	67	0.87(0.85,0.89),I^2^=75.37%,p<0.001	0.87(0.85,0.89),I^2^=81.77%,p<0.001	6.9(5.8,8.2)	0.15(0.12,0.18)	47(34,64)	0.94(0.91,0.95)
**Subgroup3: specimen**							
Serum	77	0.84(0.81,0.87),I^2^=91.62%,p<0.001	0.84(0.81,0.86),I^2^=82.12%,p<0.001	5.2(4.5,6.1)	0.19(0.15,0.23)	28(21,38)	0.91(0.88,0.93)
Plasma	41	0.79(0.75,0.82),I^2^=84.08%,p<0.001	0.85(0.82,0.88),I^2^=80.03%,p<0.001	5.3(4.2,6.8)	0.25(0.20,0.30)	22(15,32)	0.89(0.86,0.91)
PBMCs/Neutrophils	7	0.80(0.75,0.84),I^2^=29.99%,p=0.20	0.79(0.75,0.83),I^2^=0.0%,p=0.54	3.8(3.1,4.7)	0.26(0.21,0.32)	15(11,21)	0.85(0.82,0.88)
Peripheral blood	9	0.89(0.82,0.94),I^2^=91.38%,p<0.001	0.90(0.81,0.95),I^2^=89.42%,p<0.001	8.5(4.3,16.9)	0.12(0.06,0.22)	72(22,236)	0.95(0.93,0.97)
**Subgroup4: source of control**							
Healthy control	110	0.83(0.80,0.85),I^2^=90.03%,p<0.001	0.84(0.82,0.86),I^2^=81.27%,p<0.001	5.3(4.6,6.1)	0.21(0.18,0.24)	25(20,33)	0.90(0.87,0.93)
Cancer-free control	24	0.83(0.78,0.87),I^2^=80.12%,p<0.001	0.86(0.82,0.89),I^2^=85.09%,p<0.001	6.0(4.6,7.8)	0.20(0.15,0.26)	30(18,48)	0.91(0.88,0.93)
BPD	9	0.77(0.67,0.85),I^2^=85.15%,p<0.001	0.87(0.83,0.90),I^2^=10.23%,p<0.001	5.8(4.2,8.0)	0.26(0.18,0.39)	22(11,43)	0.89(0.86,0.92)
**Subgroup5: histology type**							
AD>50%	74	0.83(0.79,0.86), I^2^=90.98%,p<0.001	0.85(0.83,0.88),I^2^=83.31%,p<0.001	5.6(4.7,6.7)	0.20(0.17,0.25)	27(20,38)	0.91(0.88,0.93)
AD<50%	48	0.82(0.79,0.85),I^2^=85.09%,p<0.001	0.84(0.81,0.87),I^2^=78.36,p<0.001	5.1(4.2,6.2)	0.21(0.18,0.26)	24(17,34)	0.90(0.87,0.92)
NSCLC	122	0.83(0.80,0.85), I^2^=89.51%,p<0.001	0.84(0.82,0.86), I^2^=78.42%,p<0.001	5.2(4.5,5.8)	0.21(0.18,0.24)	25(20,31)	0.90(0.87,0.92)
Lung cancer(mixed)	10	0.87(0.80,0.92), I^2^=81.47%,p<0.001	0.85(0.77,0.95) I^2^=92.35%,p<0.001	6.0(3.8,9.5)	0.15(0.10,0.23)	40(20,78)	0.93(0.90,0.95)
**Subgroup6: stage**							
I/II>0.6	68	0.84(0.80,0.87),I^2^=91.70%,p<0.001	0.86(0.83,0.88),I^2^=82.26%,p<0.001	6.0(5.0,7.2)	0.19(0.15,0.24)	32(22,45)	0.92(0.89,0.94)
I/II<0.4	57	0.82(0.79,0.84),I^2^=84.74%,p<0.001	0.83(0.80,0.86),I^2^=78.01%,p<0.001	4.8(4.1,5.7)	0.22(0.19,,0.26)	22(16,29)	0.89(0.86,0.92)
Stage I/II patients	43	0.81(0.77,0.84),I^2^=83.15%,p<0.001	0.82(0.78,0.85),I^2^=83.35%,p<0.001	4.5(3.7,5.5)	0.23(0.19,0.28)	19(14,27)	0.88(0.85,0.91)
Stage I patients	20	0.80(0.75,0.84),I^2^=71.63%,p<0.001	0.81(0.76,0.86),I^2^=78.62%,p<0.001	4.3(3.2,5.8)	0.25(0.19,0.32)	18(10,30)	0.88(0.84,0.90)
**Subgroup7: sample size**							
>150	50	0.80(0.76,0.84),I^2^=92.78%,p<0.001	0.85(0.82,0.87),I^2^=84.64%,p<0.001	5.3(4.4,6.5)	0.23(0.19,0.28)	23(16,33)	0.90(0.87,0.92)
<150	84	0.84(0.81,0.87),I^2^=83.53%,p<0.001	0.84(0.81,0.86), I^2^=75.76%,p<0.001	5.3(4.5,6.2)	0.18(0.15,0.22)	28(21,38)	0.91(0.88,0.93)
**Subgroup8: publication year**							
>2015	80	0.82(0.79,0.85),I^2^=90.35%,p<0.001	0.85(0.83,0.88),I^2^=85.43%,p<0.001	5.6(4.7,6.7)	0.21(0.18,0.25)	27(20,36)	0.91(0.88,0.93)
<2015	54	0.84(0.80,0.88),I^2^=86.81%, p<0.001	0.83(0.80,0.85),I^2^=64.91%,p<0.001	4.9(4.1,5.7)	0.19(0.15,0.24)	26(18,36)	0.90(0.87,0.92)
**Subgroup9: specific miRNA**							
hsa-miR-21-5p	13	0.71(0.64,0.78),I^2^=82.09%, p<0.001	0.77(0.69,0.83),I^2^=77.44%, p<0.001	3.0(2.3,4.0)	0.38(0.30,0.48)	8(5,12)	0.80(0.77,0.84)
hsa-miR-155-5p	6	0.81(0.67,0.90),I^2^=84.93%, p<0.001	0.74(0.67,0.81),I^2^=50.45%, p=0.07	3.2(2.3,4.3)	0.26(0.14,0.46)	12(5,27)	0.81(0.77,0.84)
hsa-miR-145-5p	6	0.74(0.61,0.83),I^2^=82.86%, p<0.001	0.69(0.56,0.79),I^2^=81.53%, p<0.001	2.4(1.5,3.7)	0.38(0.23,0.63)	6(2,15)	0.77(0.73,0.81)

SEN=sensitivity, SPE= specificity, PLR= positive likelihood ratio, NLR= negative likelihood ratio, DOR= diagnostic odds ratio, AUC = area under the curve, CI= confidence interval, PBMCs =peripheral blood mononuclear cells, BPD= benign pulmonary disease, AD= adenocarcinoma.

### Subgroup analyses

**Figure 4:**
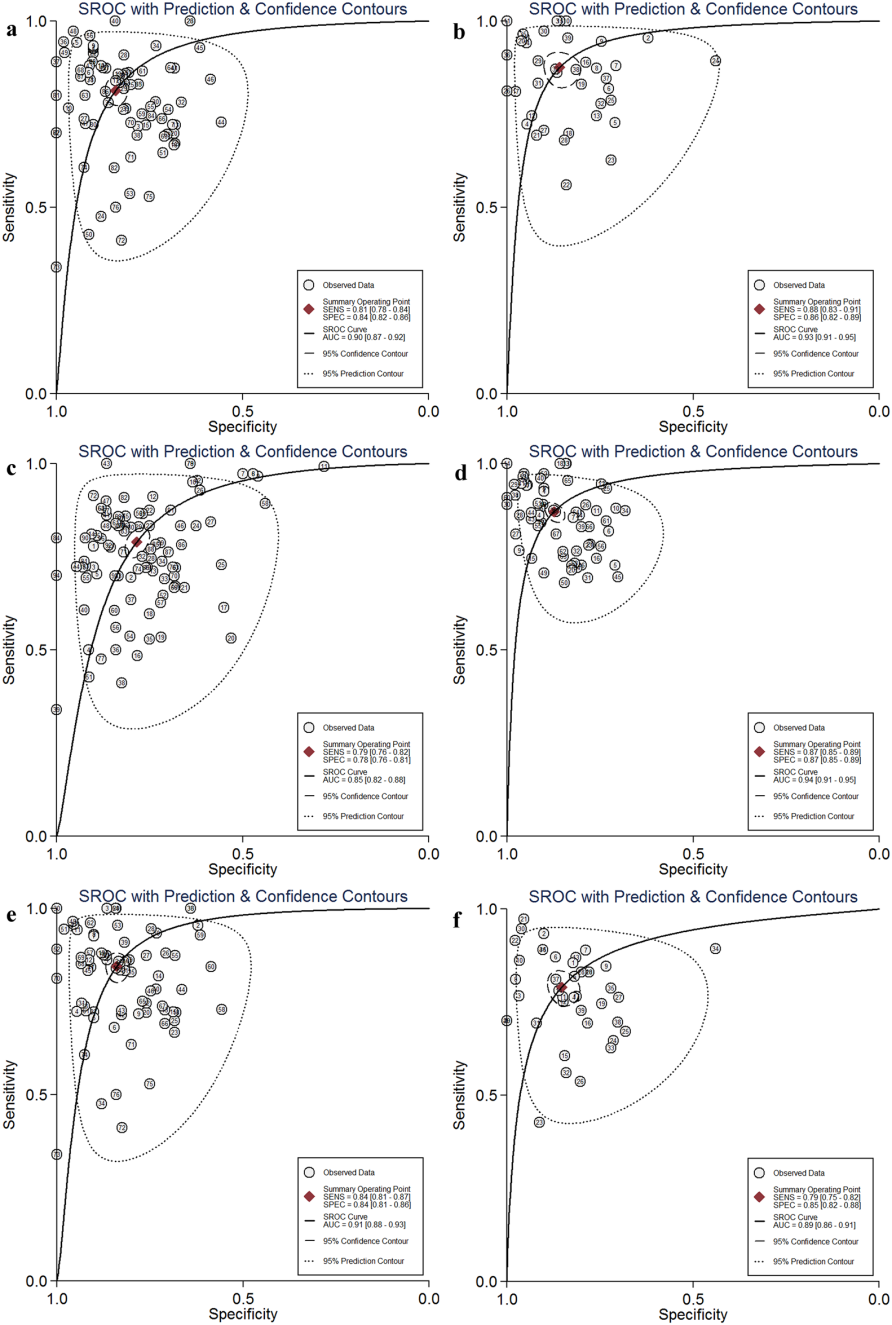
Subgroup analysis of the summary receiver operator characteristic (SROC) curves of the miRNA test for the diagnosis of lung cancer patients **(a)** Asian population, **(b)** Caucasian, **(c)** single miRNA, **(d)** multiple miRNA, **(e)** serum, **(f)** plasma).

Various subgroup analyses (race, miRNA profiling, specimen, TNM stage, histology type, source of control, sample size and publication year) were also done. The main results of subgroup analyses are summarized in Table 1 and Figure 4.

#### Race

In the subgroup analysis according to race, It seemed the circulating miRNAs may yield higher diagnostic performance in Caucasian than in Asian and mixed population (SEN: 0.88 vs 0.81 vs 0.76, SPE: 0.86 vs 0.84 vs 0.82, PLR: 6.2 vs 5.1 vs 4.3, NLR: 0.14 vs 0.22 vs 0.29, DOR: 43 vs 23 vs 15, and AUC: 0.93 vs 0.90 vs 0.86 for Caucasian, Asian and mixed population respectively) (Table 1). Figure 4a and 4b illustrate the SROC curves of diagnostic role of circulating miRNAs in Asian and Caucasian population respectively.

#### MiRNA profiling

We found combined miRNAs may lead higher diagnostic accuracy when compared with single miRNAs (SEN: 0.87 vs 0.79, SPE: 0.87 vs 0.78, PLR: 6.9 vs 3.7, NLR: 0.15 vs 0.27, DOR: 47 vs 14, and AUC: 0.94 vs 0.85 for multiple (n≥2) and single (n=1) miRNAs respectively) (Table 1 and Figure 4c and 4d).

#### Specimen

The specimen used in our included studies could be categorized as serum, plasma, peripheral blood mononuclear cells (PBMCs)/neutrophils and peripheral blood. 77 studies applied miRNAs detection in serum, which was the most widely used material. 41 studies applied plasma to detect miRNAs. The diagnostic performance of miRNAs in serum and plasma were good, with sensitivity of 0.84 and 0.79, specificity of 0.84 and 0.85, PLR of 5.2 and 5.3, NLR of 0.19 and 0.25, DOR of 28 and 22, and AUC of 0.91and 0.89 (Figure 4e and 4f). Detecting miRNAs in PBMCs/neutrophils and peripheral blood also had good performance to distinguish lung cancer patients from controls (Table 1).

#### TNM stage

**Figure 5:**
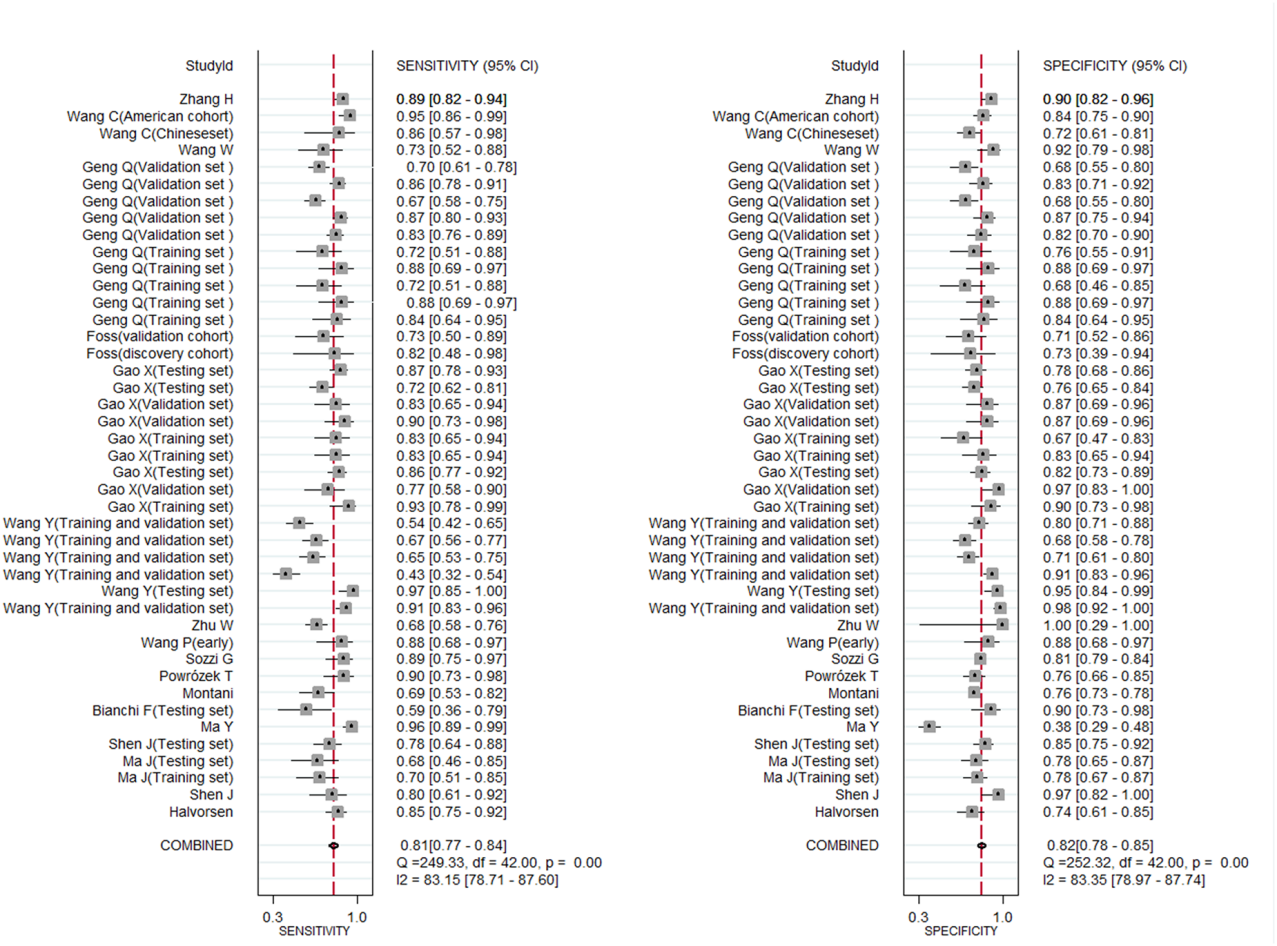
Forest plots of sensitivity and specifcity for circulating miRNAs in the diagnosis of stage I/II lung cancer

**Figure 6:**
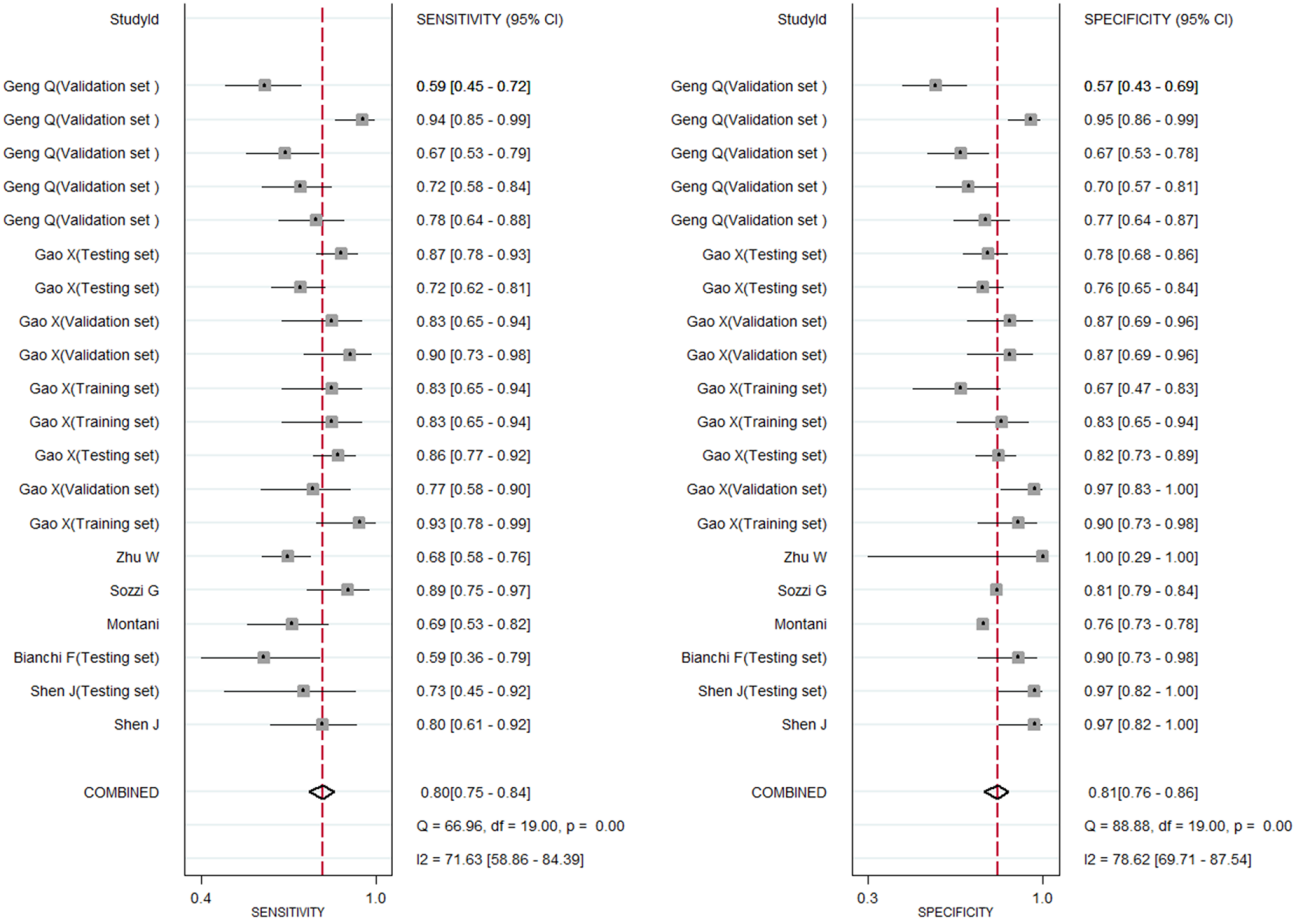
Forest plots of sensitivity and specifcity for circulating miRNAs in the diagnosis of stage I lung cancer

Firstly, we divided studies into two groups according to the percentage of stage I/II in overall lung cancer patients and 0.6 was selected as cutoff value. We found circulating miRNAs in patients with stage I/II≥0.6 and <0.4 all yielded good diagnostic performance. In addition, we further explored the diagnostic role of miRNAs in early stage lung cancer. We found the diagnostic accuracy of stage I/II and an additional stage I group were just similar to overall stage lung cancer (for stage I/II patients, the sensitivity, specificity, PLR, NLR, DOR and AUC were 0.81, 0.82, 4.5, 0.23, 19 and 0.88 respectively; for stage I patients, they were 0.80, 0.81, 4.3, 0.25, 18 and 0.88 respectively) (Figures 5 and 6). This suggested circulating miRNAs could distinguish all stage lung cancer from controls correctly (Table 1).

#### Source of controls

The controls of 110 studies were from healthy population, and 24 studies included cancer-free population as controls. Among 24 studies, nine were benign lung disease (BPD). Studies with healthy controls had a pooled sensitivity of 0.83, specificity of 0.84, PLR of 5.3, NLR of 0.21, DOR of 25 and the AUC of 0.90. For studies with cancer-free controls, the pooled sensitivity was 0.83, specificity was 0.86, PLR was 6.0, NLR was 0.20, DOR was 30 and the AUC was 0.91. In BPD controls, the sensitivity was 0.77, specificity was 0.87, PLR was 5.8, NLR was 0.26, DOR was 22 and the AUC was 0.89. Circulating miRNAs could not only screen lung cancer from healthy population, but also distinguish lung cancer from cancer-free patients (Table 1).

#### Histology type, sample size and publication year

74 studies investigated mostly (≥50%) with adenocarcinoma (AD). We performed subgroup analysis according to the percentage of AD≥50% and <50%, we found both groups had similar diagnostic accuracy. Only two studies evaluated the SCLC, as a result, we failed to conduct subgroup analysis in SCLC. The subgroup analysis was done in NSCLC and lung cancer (mixed type). The result suggested the diagnostic role of miRNAs in both groups was good (SEN: 0.83 vs 0.87, SPE: 0.84 vs 0.85, PLR: 5.2 vs 6.0, NLR: 0.21 vs 0.15, DOR: 25 vs 40, and AUC: 0.90 vs 0.93 for NSCLC and lung cancer, respectively) (Table 1).

The diagnostic accuracy was similar in the subgroup analyses in sample size and publication year (Table 1).

### Meta-regression analyses and publication bias

**Figure 7:**
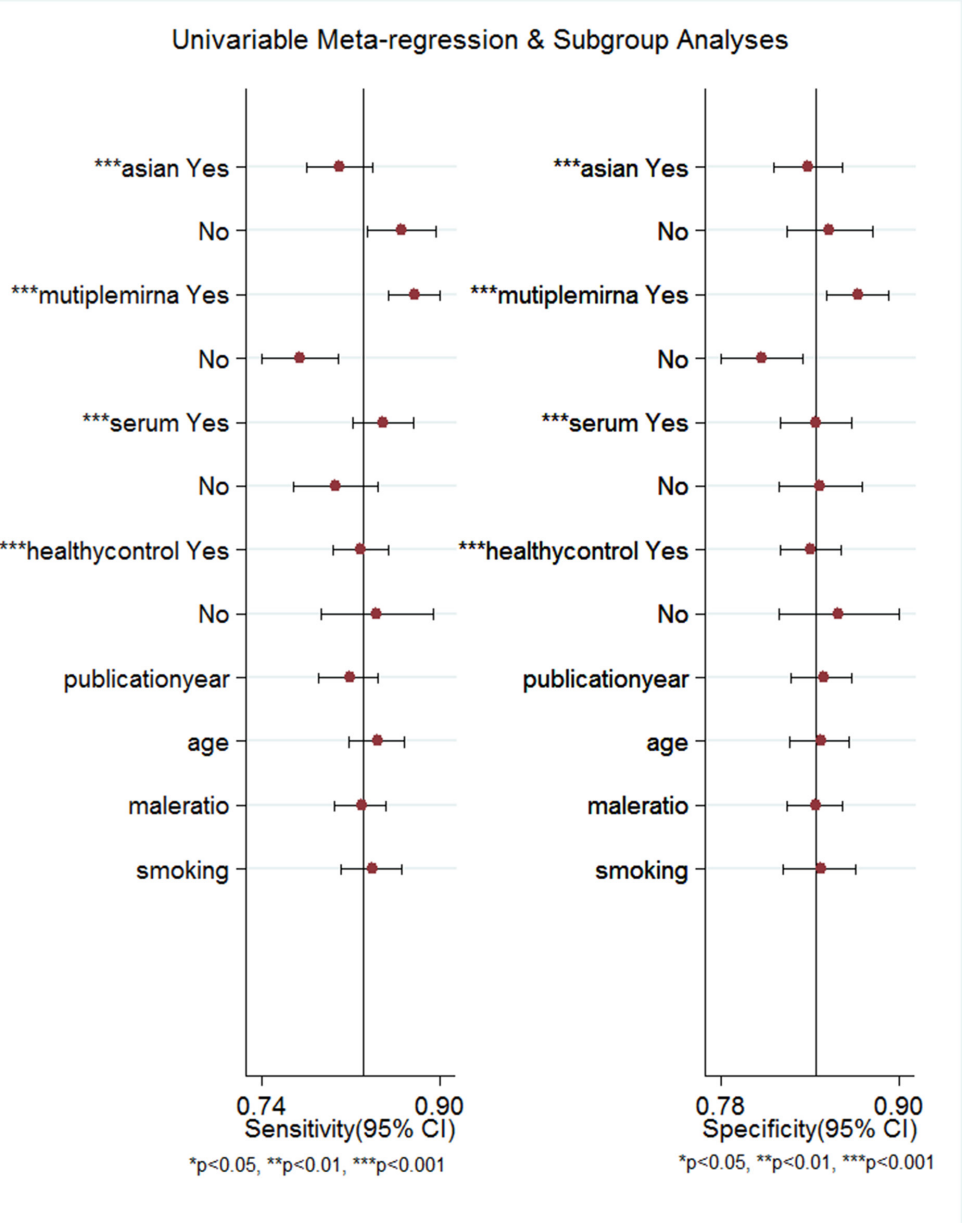
Forest plots of multivariable meta-regression analyses for sensitivity and specificity

**Figure 8:**
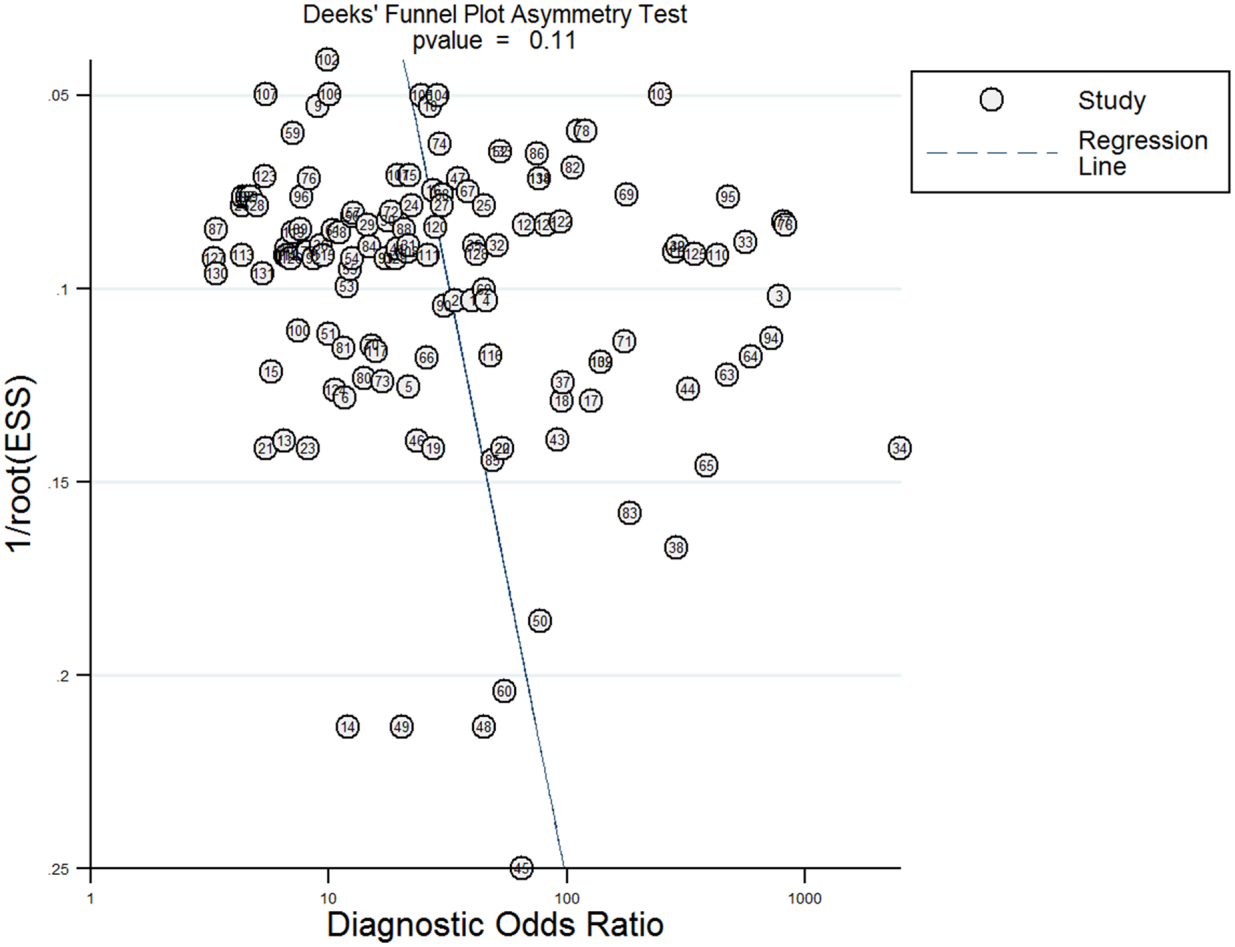
Deeks’ linear regression test of funnel plot asymmetry

Meta-regression analyses were performed to analyze the potential sources of inter-study heterogeneity. The analysis suggested race (P<0.001), miRNA profiling (P<0.001), specimen (P<0.001), and the source of control (P<0.001) maybe the main sources of heterogeneity (Figure 7). As shown in Figure 8, no significant publication bias was detected by Deeks’funnel plot asymmetry test (P=0.11).

### Potential miRNAs as circulating diagnostic biomarkers identified from literature

To identify potential circulating miRNA biomarkers from literature, differentially expressed circulating miRNAs in a consistent direction of change reported by at least two studies were sorted. Table 2 lists the potential miRNAs fulfilled the criteria above. A total of 42 miRNAs were reported by at least two studies in a consistent direction. Except three miRNAs have conflict results (hsa-miR-145-5p, hsa-miR-486-5p and hsa-miR-125a-5p), other 39 miRNAs have aconsistent results. Among 39 miRNAs, 20 miRNAs were upregulated, and the reaming 19 miRNAs were downregulated. Hsa-miR-21-5p was reported upregulated by 13 studies, followed by hsa-miR-223-3p, which was reported by seven studies as upregulated miRNA. Six studies reported two miRNAs (hsa-miR-155-5p and hsa-miR-126-3p); five studies reported two miRNAs (hsa-miR-210-5p and hsa-miR-20a-5p); four studies reported two miRNAs (hsa-miR-182-5p and hsa-miR-148b-3p); and three studies reported five miRNAs (hsa-miR-205-5p, hsa-miR-25-5p, hsa-miR-19b-3p, hsa-let-7a and hsa-let-7d). Also, we tried to examine the individual specificity and sensitivity of these specific miRNAs by meta-analysis. Only three miRNAs (miR-21-5p, miR-155-5p and miR-145-5p) were available for analysis, the analysis showed the diagnostic performance of these three miRNAs were good (SEN: 0.71 vs 0.81 vs 0.74, SPE: 0.77 vs 0.74 vs 0.69, PLR: 3.0 vs 3.2 vs 2.4, NLR: 0.38 vs 0.26 vs 0.38, DOR: 8 vs 12 vs 6, and AUC: 0.80 vs 0.81 vs 0.77 for miR-21-5p, miR-155-5p and miR-145-5p, respectively) (Table 1).

**Table 2:** The differentially expressed miRNAs with a consistent direction reported in at least two studies

miRNAs	Accession	Mature sequence	Source	Direction of expression	Studies	Reference
hsa-miR-21-5p	MIMAT0000076	uagcuuaucagacugauguuga	Literature	↑	13	11,19,25,32, 40,47,48,53,60, 64,66,72,74
hsa-miR-223-3p	MIMAT0000280	cguguauuugacaagcugaguu	Microarray,literature	↑	7	12,13,19,34, 39,46,69
hsa-miR-155-5p	MIMAT0000646	uuaaugcuaaucgugauaggggu	Literature	↑	6	11,17,19,46, 53,70
hsa-miR-210-5p	MIMAT0026475	agccccugcccaccgcacacug	Literature	↑	5	33,47,48,58,72
hsa-miR-20a-5p	MIMAT0000075	uaaagugcuuauagugcagguag	Microarray,literature	↑	5	13,15,19,46,69
hsa-miR-182-5p	MIMAT0000259	uuuggcaaugguagaacucacacu	Literature	↑	4	11,47,48,72
hsa-miR-145-5p	MIMAT0000437	guccaguuuucccaggaaucccu	Microarray,literature	↑	4	13,19,57,69
				↓	1	53
hsa-miR-205-5p	MIMAT0000266	uccuucauuccaccggagucug	Microarray,literature	↑	3	21,25,67
hsa-miR-25-5p	MIMAT0004498	aggcggagacuugggcaauug	Microarray	↑	3	13,24,67
hsa-miR-19b-3p	MIMAT0000074	ugugcaaauccaugcaaaacuga	Microarray	↑	3	35,37,74
hsa-miR-197-5p	MIMAT0022691	cggguagagagggcagugggagg	Literature	↑	2	11,70
hsa-miR-29a-3p	MIMAT0000086	acugauuucuuuugguguucag	Microarray	↑	2	12,39
hsa-miR-140-5p	MIMAT0000431	cagugguuuuacccuaugguag	Microarray	↑	2	12,39
hsa-miR-221-3p	MIMAT0000278	agcuacauugucugcuggguuuc	Microarray	↑	2	13,74
hsa-miR-222-3p	MIMAT0000279	agcuacaucuggcuacugggu	Microarray	↑	2	13,34
hsa-miR-574-5p	MIMAT0004795	ugagugugugugugugagugugu	Microarray	↑	2	16,24
hsa-miR-324-3p	MIMAT0000762	acugccccaggugcugcugg	Microarray	↑	2	18,24
hsa-miR-200b-3p	MIMAT0000318	uaauacugccugguaaugauga	Microarray	↑	2	21,41
hsa-miR-125b-5p	MIMAT0000423	ucccugagacccuaacuuguga	Microarray,literature	↑	2	21,38
hsa-miR-1244	MIMAT0005896	aaguaguugguuuguaugagaugguu	Microarray	↑	2	56,76
hsa-miR-183-5p	MIMAT0000261	uauggcacugguagaauucacu	Literature	↑	2	67,72
hsa-miR-126-3p	MIMAT0000445	ucguaccgugaguaauaaugcg	Microarray,literature	↓	6	12,24,46,47, 56,72
hsa-miR-148b-3p	MIMAT0000759	ucagugcaucacagaacuuugu	Microarray,literature	↓	4	12,29,39,64
hsa-miR-486-5p	MIMAT0002177	uccuguacugagcugccccgag	Microarray	↓	4	12,40,47,48
			Literature	↓	2	31,58
hsa-miR-125a-5p	MIMAT0000443	ucccugagacccuuuaaccuguga	Literature	↓	2	56,73
			Literature	↑	1	57
hsa-let-7a	MIMAT0000062	ugagguaguagguuguauaguu	Microarray,literature	↓	3	12,23,24
hsa-let-7d	MIMAT0000065	agagguaguagguugcauaguu	Microarray	↓	3	12,24,39
hsa-miR-328-5p	MIMAT0026486	gggggggcaggaggggcucaggg	Microarray	↓	2	12,39
hsa-miR-191-5p	MIMAT0000440	caacggaaucccaaaagcagcug	Microarray	↓	2	12,39
hsa-miR-92a-3p	MIMAT0000092	uauugcacuugucccggccugu	Microarray	↓	2	12,39
hsa-miR-484	MIMAT0002174	ucaggcucaguccccucccgau	Microarray	↓	2	12,39
hsa-miR-22-3p	MIMAT0000077	aagcugccaguugaagaacugu	Microarray	↓	2	12,24
hsa-miR-331-3p	MIMAT0000760	gccccugggccuauccuagaa	Microarray	↓	2	12,39
hsa-miR-30c	MIMAT0000244	uguaaacauccuacacucucagc	Microarray	↓	2	12,39
hsa-miR-98-5p	MIMAT0000096	ugagguaguaaguuguauuguu	Microarray	↓	2	12,24
hsa-miR-374a	MIMAT0000727	uuauaauacaaccugauaagug	Microarray	↓	2	12,39
hsa-miR-30b	MIMAT0000420	uguaaacauccuacacucagcu	Microarray	↓	2	12,39
hsa-let-7c	MIMAT0000064	ugagguaguagguuguaugguu	Literature	↓	2	14,24
hsa-let-7e	MIMAT0000066	ugagguaggagguuguauaguu	Microarray	↓	2	24,73
hsa-let-7f	MIMAT0000067	ugagguaguagauuguauaguu	Microarray,literature	↓	2	24,46
hsa-miR-195	MIMAT0000461	uagcagcacagaaauauuggc	Microarray,literature	↓	2	24,50
hsa-miR-29b-3p	MIMAT0000100	uagcaccauuugaaaucaguguu	Literature	↓	2	35,37

## DISCUSSION

The development of suitable noninvasive biomarkers is important for the diagnosis of lung cancer. The pooled data in this meta-analysis showed circulating miRNAs had a sensitivity of 0.83 (95% CI 0.80-0.85) and specificity of 0.84 (95% CI 0.82-0.86), with corresponding PLR 5.3 (95% CI: 4.7-6.0) and NLR 0.20 (95% CI: 0.18-0.23). The pooled DOR was 26 (95% CI: 21-33) and the AUC was 0.90 (95% CI: 0.88-0.93). In various subgroup analyses, we found the diagnostic accuracy in each subgroup was very high, suggesting miRNA might be potential biomarker to discriminate lung cancer from controls.

Previous meta-analyses had explored the diagnostic role of miRNA in lung cancer [76–78]. However, our study showed the following advancements when compared with previous works. Firstly, as blood is very common and effective in lung cancer screening and to avoid potential bias caused by specimen, we only focused on circulating miRNAs, but previous works focused on not only blood but also sputum and exhaled breath condensate. It was our first time to comprehensively evaluate the diagnostic role of circulating miRNAs in lung cancer. Secondly, our analysis included 65 articles including 134 studies (6,919 patients with lung cancer and 7,064 controls). The sample size included in the studies by Chen [76], Guo [77] and Wang et al. [78] were 2,623, 3,801 and 3,703 respectively. Our sample size was larger than previous works; as a result, our analysis was more robust and reliable. Besides, the pooled data in this meta-analysis suggested the diagnostic role of circulating miRNA was higher than most recent study by Chen [76], Guo [77] and Wang et al. [78] (with sensitivity of 0.78, 0.76 and 0.78; specificity of 0.80, 0.77 and 0.80; AUC of 0.86, 0.83 and 0.86 in blood-based subgroups in the studies by Chen, Guo and Wang et al. respectively). This was because we included more studies than previous studies. At last, various subgroup analyses such as race, miRNA profiling, specimen, source of control, histology type, stage, sample size and publication year were done in our analysis to provide more information and give a comprehensive insight on the diagnostic role on circulating miRNAs in lung cancer. All these subgroup analyses suggested circulating miRNAs had good diagnostic performance.

The results of subgroup analyses in our study may give some useful information to clinical practice. We found combined miRNAs could yield higher diagnostic performance than single miRNAs. Single miRNAs with good diagnostic performance should be combined to yield higher diagnostic efficacy [49]. Serum was widely used in detecting miRNAs, and miRNAs in serum had good diagnostic value in the diagnosis of lung cancer. Serum was widely used in clinical detection such as blood biochemical tests and protein marker detection. As a result, detecting miRNAs in serum was more convenience for clinical practice [79, 80]. Also, combined miRNAs with serum protein markers, such as cytokeratin 19 fragment (CYFRA21-1), carcinoembryonic antigen (CEA), neuron-specific enolase (NSE), cancer-associated antigen (CA) 125 and CA 19-9 maybe more effective in the diagnosis of lung cancer [58, 81]. We also conducted subgroup analysis based on the source of controls; the source of controls could be mainly divided into healthy population and cancer-free controls. In cancer-free controls, we could also extract a subgroup of BPD (such as pneumonia and benign pulmonary nodules). We found the diagnostic accuracy of miRNAs in these subgroups were similar, suggesting circulating miRNAs could not only serve as screening biomarkers but also could serve as biomarkers to distinguish lung cancer from BPD. Also, the diagnostic value of miRNAs in early stage lung cancer (stage I and stage I/II) is also good when compared with overall patients, suggesting circulating miRNAs were ideal biomarkers in all stage of lung cancers.

The circulating miRNAs may yield higher diagnostic performance in Caucasian than in Asian and mixed population, the racial disparities was found. These differences could be attributed to environmental, diet and life-style [82]. All these changes have been shown to affect the expression of miRNAs. For example, these factors could be affected the expression of miRNAs by methylation of CpG islands in their promoters [82, 83]. Another possible reason was the number of studies included for analysis was different in each groups. In Asian subgroups, 86 studies were included for analysis, but in Caucasian and mixed populations, only 39 and nine studies were included. The studies included for analysis in Asian population was generally two times more than Caucasian population and even more than mixed population.

We think the major obstacle was no uniform circulating miRNA biomarkers used in each study. Most studies selected miRNAs by microarray [12, 13, 18, 21, 22, 24, 26–28, 34–37, 39, 41, 42, 46–49, 52, 55, 59, 61, 64, 67, 74]. Some identified miRNAs by literature [11, 14–16, 19, 23, 25, 29–33, 40, 43–45, 50, 51, 53, 54, 56–58, 60, 62, 63, 65, 66, 68–73, 75, 84]. To give an insight to future studies, we listed the potential miRNAs reported by literature that could serve as circulating biomarkers. We found some upregulated miRNAs such as hsa-miR-21-5p, hsa-miR-223-3p, hsa-miR-155-5p, hsa-miR-210-5p, hsa-miR-205-5p, hsa-miR-25-5p and hsa-miR-19b-3p; some downregulated miRNAs such as hsa-miR-126-3p, hsa-miR-148b-3p, hsa-let-7a and hsa-let-7d, might be served as potential biomarkers. However, we also found some miRNAs such as hsa-miR-486-5p and hsa-miR-125a-5p were reported in inconsistent direction. Four studies found that hsa-miR-486-5p was reduced in peripheral blood of lung cancer patients [12, 40, 47, 48], while others two reported that it was up-regulation in lung cancer patients [31, 58]. The studies by Wang et al. [56] and Zhu et al. [73] reported that hsa-miR-125a-5p was downregulated in serum of lung cancer patients, but another study [57] found it was upregulated in serum. The contradictory results may be caused by inconsistent cutoff values applied by different studies and the potential bias existed in sample selection in each study. These miRNAs with conflicting results should be validated rigorously to verify whether they were suitable to serve as diagnostic biomarkers in lung cancer. In our study, we identified several miRNAs that might serve as potential biomarkers, it would be helpful for further studies to validate them; also, more works should be done in identifying more useful miRNAs and uniform their cutoff values to make them more feasible and effective in clinical practice.

We think the major limitation of our study was high heterogeneity exist in our analysis. Meta-regression analysis was done to explore the potential sources of heterogeneity between included studies. We found race, miRNA profiling, specimen, and the source of control might be the major cause of heterogeneity. Except that, no uniform cutoff values and certain miRNAs were applied in each study. The methodologies for an accurate absolute quantification of miRNAs and selected certain miRNAs limited the cross-comparison between studies performed by different laboratories.

In summary, the present study suggests that circulating miRNAs could serve as non-invasive diagnostic biomarkers for all stage lung cancers. They could not only screen lung cancer from healthy population, but also have a role in distinguishing lung cancer from cancer-free patients. Particularly, combinations of miRNAs are more complete indicators than individual miRNAs. Further subgroup analysis also indicated that serum might serve as the ideal sample specimen for the detecting miRNAs in the diagnosis of lung cancers. We also identified a panel of miRNAs such as miR-21-5p, miR-223-3p, miR-155-5p and miR-126-3p that might serve as potential biomarkers for lung cancer. However, the clinical application of miRNA profiling for lung cancer detection still needs further validation by future studies.

## MATERIALS AND METHODS

### Literature search

A comprehensive literature search was conducted in the databases of Medline, PubMed, EMBASE and Web of Science. The last search time was May 31, 2017. The following terms and combinations were used to identify studies: “microRNA”, “miRNA”, “lung cancer” and “lung neoplasm”. Furthermore, references of retrieved articles and reviews were manually screened for additional studies.

### Inclusion and exclusion criteria

The inclusion criteria were applied to identify the eligible studies: (1) human-based investigations; (2) articles with full texts published in English and Chinese; (3) all lung cancer cases should be confirmed by pathology; (4) miRNA expression level was detected in blood (serum, plasma, peripheral whole blood and leukocyte in peripheral blood); (5) studies regarding the diagnostic potential of circulating miRNAs and lung cancer and provided sufficient data to extract true positive (TP), false positive (FP), true negative (TN), and false negative (FN). The exclusion criteria were as the follows: (1) publications unrelated to the diagnostic values of circulating miRNAs for lung cancer; (2) studies with duplicate data reported in other studies; (3) letters, editorials, case reports or reviews.

### Data extraction and quality assessment

The following baseline characteristics and data were extracted: the name of first author, year of publication, country, ethnicity, and sample size; baseline characteristics of participants (age, the percentage of male, smoking status, histology, and stage of lung cancer cases, the source of control, sample species and microRNA profiling). Also, data required for diagnostic meta-analysis (TP, FP, TN and FN) were also extracted from included studies. The quality of each eligible study was assessed by the revised Quality Assessment of Diagnostic Accuracy Studies tool [85]. The data extraction and examination, quality assessments were conducted mainly by two investigators independently. Disagreements between the investigators were resolved by discussion among all authors until reach a consensus.

### Extraction of potential circulating miRNAs as diagnostic biomarkers

To identify potential circulating miRNA biomarkers from literature, differentially expressed circulating miRNAs in a consistent direction of change reported by at least two studies were sorted. These miRNAs were analyzed individually or as one of the miRNAs panels. Some studies reported the TP, FP, TN and FN, but some studies did not report them. To evaluate the diagnostic accuracy of these specific miRNAs, the pooled sensitivity and specificity were performed if the data were available for analysis.

### Statistical analysis

STATA 12.0 software (StataCorp, College Station, TX, USA) is used to perform all statistical analyses. We used the bivariate random-effects meta-analysis model to calculate the pooled sensitivity (SEN) [TP/(TP+FN)], specificity (SPE) [TN/(TN+FP)], positive likelihood ratio (PLR) [(sensitivity/(1−sensitivity)], negative likelihood ratio (NLR) [(1−specificity)/specificity)] and diagnostic odds ratio (DOR) [PLR/NLR] with their corresponding 95 % confidence intervals (CIs). The summary receiver operator characteristic (SROC) curve was plotted based on the sensitivity and specificity (sensitivity as the vertical axis, specificity as the horizontal axis). The area under the curve (AUC) was also calculated to evaluate the diagnostic accuracy of miRNA in discriminating lung cancer patients from controls [86, 87]. The Q test and I^2^ test was conducted to analyze the heterogeneity between studies. A P value less than 0.10 for Q test or I^2^ more than 50% indicated that there is substantial between-study heterogeneity [88]. To further explore the potential sources of heterogeneity, subgroup analyses and meta-regression were performed according to the characteristics of the included studies. Publication bias was assessed by using Deek's funnel plot asymmetry test (P<0.10 indicating statistically significant) [89].

## SUPPLEMENTARY MATERIALS FIGURE AND TABLE





## Abbreviations

AD: adenocarcinoma
AUC: area under the curve
BPD: benign lung disease
CI: confidence intervals
CT: computed tomography
DOR: diagnostic odds ratio
FN: false negative
FP: false positive
NLR: negative likelihood ratio
NSCLC: non-small-cell lung cancer
PBMCs: peripheral blood mononuclear cells
PLR: positive likelihood ratio
PRISMA: preferred reporting items for meta-analyses
SCLC: small cell lung cancer
SEN: sensitivity
SPE: specifcity
SROC: summary receiver operating characteristic
TN: true negative
TP: true positive
