# Supplementary material for: The clinical use of circulating microRNAs as non-invasive diagnostic biomarkers for lung cancers
Source: Oncotarget. 2017 Oct 4;8(52):90197–214. doi: 10.18632/oncotarget.21644 (PMC5685742; doi:10.18632/oncotarget.21644)
Supplement: Supplementary file 1 [file oncotarget-08-90197-s001.pdf]

The clinical use of circulating microRNAs as non-invasive diagnostic biomarkers for lung cancers

SUPPLEMENTARY MATERIALS

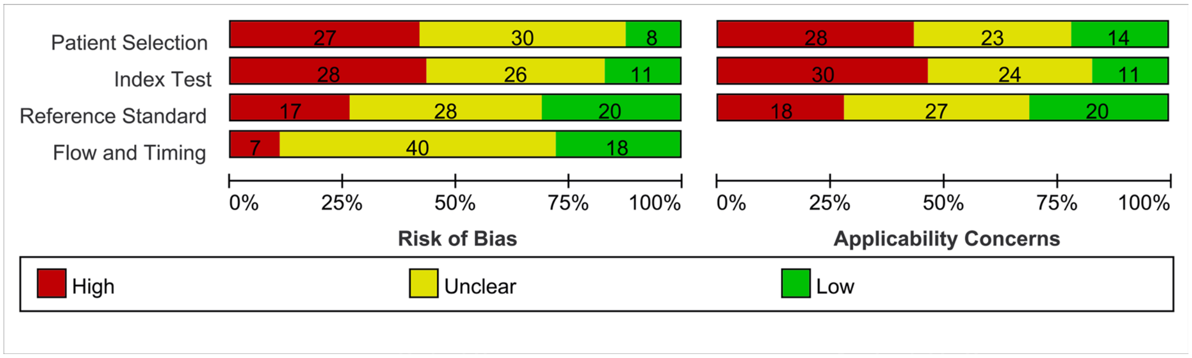

Supplementary Figure 1: Methodological quality graph of included studies.

Supplementary Table 1: The main characteristics of included studies.

See Supplementary File 1
