# Supplementary material for: The clinical use of circulating microRNAs as non-invasive diagnostic biomarkers for lung cancers
Source: Oncotarget. 2017 Oct 4;8(52):90197–214. doi: 10.18632/oncotarget.21644 (PMC5685742; doi:10.18632/oncotarget.21644)
Supplement: Supplementary file 2 [file oncotarget-08-90197-s002.docx]

**Supplementary Table 1: The main characteristics of included studies**

| **Study,year** | **Ehtnicity(Country)** | **No. of cases(case**  **/control)** | **Mean/medan age** | **Male Ratio** | **Smoking status (Yes Ratio)** | **Histology(AD Ratio)** | **Stage(I/II Ratio)** | **Source of controls** | **Sample** | **MicroRNA profiling** |
| --- | --- | --- | --- | --- | --- | --- | --- | --- | --- | --- |
| Abd-El-Fattah,2013 | Asian(Egypt) | 65/37 | 54.1/50.1 | 0.57/0.58 | NR | LC(1.00) | IV(1.00) | HC | Serum | miR-21,155,182,197 |
| Bianchi F.2011 | Caucasian(Italy) | 25/39 | 58 | 0.73 | 1/1 | AD(1.00) | I-IV(>0.84) | CF | Serum | miRNA panel(34) |
|  |  | 34/30 | 60 | 0.672 | 1/1 | NSCLC(0.65) | I-IV(>0.65) | CF | Serum | miRNA panel(34) |
| Chen X 2012 | Asian(China) | 200/110 | 59.6/57.9 | 0.67/0.69 | 0.62/0.47 | NSCLC(0.62) | I-IV(0.36) | HC | Serum | miRNA panel(10) |
|  |  | 200/110 | 59.5/58.7 | 0.78/0.72 | 0.74/0.59 | NSCLC(0.54) | I-IV(0.44) | HC | Serum | miRNA panel(10) |
| Dou H,2015 | Asian(China) | 120/360 | 63.2/NR | 0.6/NR | NR | NSCLC(0.47) | I-IV(0.57) | HC | Serum | let-7c,miR-152 |
| Fan L,2015 | Asian(China) | 98/58 | 60.5/58.1 | 0.54/0.53 | 0.44/0.41 | NSCLC(0.81) | I-IIIb(>0.71) | HC | Serum | miRNA panel(miR-15,16,20) |
|  |  | 70/54 | 59.7/58.0 | 0.60/0.54 | 0.4/0.37 | NSCLC(0.8) | I-IIIb(>0.70) | HC | Serum | miRNA panel(miR-15,16,21) |
| Foss,2011 | Caucasian(USA) | 11/11 | 65/64 | 0.91/1.00 | 1/0.91 | NSCLC(0.54) | I-II(1.00) | HC | Serum/plasma | miR-574-5p, 1254 |
|  |  | 22/31 | 62/62 | 0.86/0.81 | 0.95/1.00 | NSCLC(0.45) | I-II(1.00) | HC | Serum/plasma | miR-574-5p, 1254 |
| Gao F,2013 | Asian(China) | 36/32 | 55.1/52.5 | 0.31/0.31 | NR | AD(1.00) | I-IV(0.39) | HC | Serum | miR-155 |
| Gao X,2016 | Asian(China) | 30/30 | 61/60 | 0.83/0.84 | NR | NSCLC(0) | I(1.00) | HC | Plasma | miR-324-3p,1285 |
|  |  | 30/30 | 63/62 | 0.93/0.90 | NR | NSCLC(0) | I(1.00) | HC | Plasma | miR-324-3p,1285 |
|  |  | 90/90 | 62/62 | 0.8/0.78 | NR | NSCLC(0) | I(1.00) | HC | Plasma | miR-324-3p,1285 |
| Geng Q,2014 | Asian(China) | 25/25 | NR | 0.60/0.48 | 0.88/0.84 | NSCLC(0.32) | I-II(1.00) | HC | Serum | miR-20a,223,21,221,145 |
|  |  | 126/60 | NR | 0.69/0.6 | 0.71/0.47 | NSCLC(0.36) | I-II(1.00) | HC | Serum | miR-20a,223,21,221,145 |
| Guo W,2015 | Asian(China) | 126/50 | 65 (44–80) | 0.64/NR | 0.63/NR | NSCLC(NR) | I-IV(0.52) | HC | Plasma | miR-204 |
| Halvorsen,2016 | Mixed(Norway) | 100/58 | 62.6/57.6 | 0.72/0.59 | 0.87/1.00 | NSCLC(0.98) | I-IV(0.79) | CF | Serum | miRNA panel (miR-125b, 200b, 203, 205, 34b, 429) |
| Hennessey,2012 | Caucasian(USA) | 55/75 | 68/66 | 0.56/0.67 | 1/0.87 | NSCLC(0.54) | I-IV(0.84) | HC | Serum | miRNA panel(miR-15a,15b,27b,301,142-3p) |
| Jeong,2011 | Asian(Korea) | 35/30 | 67/60 | 0.80/0.53 | 0.83/0.87 | NSCLC(0.51) | I-IV(0.17) | HC | PB | let-7a |
| Keller,2009 | Caucasian(Germany) | 17/19 | NR | 0.53/0.37 | NR | NSCLC(0.41) | I-III(0.76) | HC | PB | miRNA panel(24) |
| Le H,2012 | Asian(China) | 82/50 | 59 (42–77) | 0.59/NR | NR | NSCLC(0.50) | I-IV(>0.55) | HC | Serum | miR-21,205,30d,24 |
| Leidinger | Caucasian(Germany) | 74/20 | 66/50 | NR | NR | NSCLC(NR) | NR | HC | PB | miRNA panel(10) |
| Leidinger | Caucasian(Germany) | 7/7 | 64/41 | 0.57/0.71 | NR | LC(0.17) | NR | HC | PB | miRNA panel(50) |
| Leidinger | Caucasian(Germany) | 28/24 | 64/68 | 0.50/0.42 | NR | NSCLC(0.39) | I-IIIb(0.86) | COPD | PB | miRNA panel(250) |
| Li L,2015 | Asian(China) | 36/20 | 56/61 | 0.69/0.60 | NR | NSCLC(0.56) | I-IV(0.5) | BPD | Serum | miR-148a,148b,152 |
| Li M,2014 | Asian(China) | 514/54 | NR | 0.52/NR | NR | NSCLC(0.56) | I-IV(0.32) | HC | Serum | miR-499 |
| Li W,2015 | Asian(China) | 11/11 | 59/55 | 0.64/0.54 | NR | NSCLC(NR) | NR | HC | Plasma | miR-486,150 |
| Li Y,2011 | Asian(China) | 20/10 | NR | 0.80/NR | NR | NSCLC(0.30) | I-IV(0.40) | HC | PB | miR-21 |
| Li Z,2013 | Asian(China) | 60/30 | 53.9/57 | 0.70/0.50 | NR | NSCLC(0.50) | I-IV(0.40) | HC | Serum | miR-210 |
| Lv S,2017 | Asian(China) | 120/120 | 59.9/59.4 | 0.52/0.51 | NR | AD(1.00) | I-III(0.87) | HC | Serum | miR-146a,222,223 |
| Ma J,2015 | Mixed(USA) | 84/69 | 64/62 | 0.66/0.67 | NR | NSCLC(0.51) | I-IV(0.52) | HC | PBMCs | miR-19b-3p,29b-3p |
|  |  | 56/64 | 65.1/61.3 | 0.70/0.78 | NR | NSCLC(0.52) | I-IV(0.46) | HC | PBMCs | miR-19b-3p,29b-3p |
| Ma J,2016 | Mixed(USA) | 82/73 | 66.3/65.7 | 0.68/0.63 | NR | NSCLC(0.52) | I-IV(0.49) | HC | Neutrophils | miR-26a-2-3p, 574-3p |
|  |  | 60/58 | 67.8/64.7 | 0.65/0.66 | NR | NSCLC(0.52) | I-IV(0.42) | HC | Neutrophils | miR-26a-2-3p, 574-3p |
| Ma J,2016 | Mixed(USA) | 68/69 | 67.2/66.2 | 0.66/0.59 | 1.00/1.00 | NSCLC(0.5) | I-IV(0.53) | BPN | PBMCs | miR-19b-3p,29b-3p |
|  |  | 56/55 | 67.0/65.3 | 0.66/0.65 | 1.00/1.00 | NSCLC(0.41) | I-IV(0.55) | BPN | PBMCs | miR-19b-3p,29b-3p |
| Ma Y,2011 | Asian(China) | 193/110 | NR | 0.72/0.57 | NR | NSCLC(NR) | I-IV(0.39) | HC | Serum | miR-125b |
| Montani F,2015 | Caucasian(Italy) | 12/12 | 55/55 | 0.75/0.75 | 1.00/1.00 | AD(1.00) | I-III(0.92) | CF | Serum | miRNA panel(13) |
|  |  | 36/972 | 57/64 | 0.69/0.70 | 1.00/1.00 | LC(0.78) | I-III(0.92) | CF | Serum | miRNA panel(13) |
| Mozzoni | Caucasian(Italy) | 54/46 | 69.1 /64.1 | 0.63/0.67 | 0.83/0.67 | NSCLC(0.68) | I-IIIb(>0.52) | BPN | Plasma | miR-21,486 |
| Nadal | Caucasian(USA) | 70/22 | 67.5/67 | 0.50/0.54 | 0.84/0.27 | NSCLC(0.86) | I-III(0.83) | CF | Serum | miR-141,200b,193b,301 |
|  |  | 84/23 | 65.5/60 | 0.51/0.35 | 0.94/0.48 | NSCLC(0.66) | I-III(0.89) | CF | Serum | miR-141,200b,193b,301 |
| Patnaik | Caucasian(USA) | 22/23 | 70.6/59.9 | 0.55/0.52 | 0.91/0.83 | AC(1.00) | I-III(0.73) | BPN,HC | PB | miRNA panel |
| Peng H | Asian(China) | 36/36 | 58.9/54.4 | 0.67/0.62 | 0.62/NR | NSCLC(0.56) | I-IV(0.64) | HC | Serum | miR-1254,574-5p,485-5p, |
|  |  | 120/71 | 60.2/57.6 | 0.72/0.62 | 0.64/NR | NSCLC(0.45) | I-IV(0.52) | HC | Perum | miR-1254,574-5p,485-5p, |
| Powrózek | Caucasian(Poland) | 90/85 | 64/57 | 0.69/0.60 | 0.94/NR | LC(0.33) | I-IV(0.32) | HC | Plasma | miR-4478,448 |
| Powrózek | Caucasian(Poland) | 90/85 | 64/57 | 0.69/0.60 | 0.94/NR | LC(0.33) | I-IV(0.32) | HC | Plasma | miR-944,3622 |
| Sanfiorenzo,2013 | Caucasian(France) | 52/30 | 65.1/68.0 | 0.75/0.73 | 0.85/0.77 | NSCLC(0.52) | I-IIIa(0.83) | COPD,HC | Plasma | miRNA panel(12) |
| Shen J,2011 | Mixed(USA) | 32/33 | 66.2/64.7 | 0.62/0.61 | NR | NSCLC(0.56) | I-IV(0.59) | BPN | Plasma | miR-21, 210,486-5p |
|  |  | 76/80 | 67.9/65.4 | 0.55/0.62 | NR | NSCLC(0.53) | I-IV(0.71) | BPN | Plasma | miR-21, 210,486-5p |
| Shen J,2011 | Mixed(USA) | 58/29 | 68/66 | 0.655/0.643 | NR | NSCLC(0.59) | I(0.52) | HC | Plasma | miR-21,126,210,182,486-5p |
| Sozzi,2014 | Caucasian(Italy) | 69/870 | 60.9/56.4 | 0.812/0.633 | 1/1 | LC(NR) | I-IV(0.54) | CF | Plasma | miRNA panel(24) |
| Su K,2016 | Asian(China) | 100/100 | NR | 0.65/0.7 | 0.85/0.86 | NSCLC(0.44) | I-III(0.57) | HC | Plasma | miR-195 |
| Sun L,2016 | Asian(China) | 99/96 | 60.7/NR | 0.687/0.604 | NR | NSCLC(0.60) | I-IV(0.60) | HC,BPN | Plasma | miR-30a |
| Tai MC,2016 | Asian(Japan) | 143/49 | 65.9/66.3 | 0.531/0.571 | 0.811/0.98 | AD(1.00) | I-IV(0.71) | HC | Serum | miRNA panel(20) |
|  |  | 110/52 | 65/65.7 | 0.564/0.577 | 0.636/0.98 | AD(1.00) | I-IV(0.71) | HC | Serum | miRNA panel(20) |
| Tang D,2013 | Asian(China) | 62/60 | 64.8/66 | 0.645/0.85 | NR/1 | NSCLC(0.64) | I-III(0.60) | HC | Plasma | miR-21,145,155 |
|  |  | 34/32 | 65.2/66.4 | 0.647/0.812 | NR/1 | NSCLC(0.59) | I-III(0.53) | HC | Plasma | miR-21,145,155 |
| Ulivi,2013 | Caucasian(Italy) | 86/24 | 68/65 | 0.651/0.625 | 0.791/0.54 | NSCLC(0.73) | I-IV(0.63) | HC | PB | miR-328 |
| Wang C,2015 | Asian(China) | 19/19 | 61.8/62.1 | 0.579/0.684 | 0.421/0.368 | NSCLC(0.47) | I-IV(0.64) | HC | Serum | miR-483-5p,193a-3p,25,214,7 |
|  | Asian(China) | 63/63 | 61.9/59.7 | 0.778/0.587 | 0.619/0.286 | NSCLC(0.41) | I-IV(0.52) | HC | Serum | miR-483-5p,193a-3p,25,214,7 |
|  | Caucasian(USA) | 108/104 | 67.2/60 | 0.481/0.446 | 0.954/0.99 | NSCLC(0.48) | I-IV(0.32) | HC,BPN | Serum | miR-483-5p,193a-3p,25,214,7 |
| Wang P,2015 | Asian(China) | 24/24 | 59.6/58.3 | 0.583/0.458 | 0.417/0.458 | NSCLC(0.72) | I-II(1) | HC | Serum | miR-125a-5p,25,126 |
|  |  | 142/111 | 61.1/59.8 | 0.606/0.522 | 0.472/0.486 | NSCLC(0.71) | I-IV(0.66) | HC | Serum | miR-125a-5p,25,126 |
| Wang R,2015 | Asian(China) | 70/70 | 64.4/63.7 | 0.614/0.614 | 0.528/0.414 | NSCLC(0.69) | NR | HC | Serum | miR-125a-5p,145,146a |
| Wang W,2016 | Asian(China) | 54/38 | 60/52 | NR | NR | NSCLC(0.48) | I-IV(0.48) | HC,BPN | Serum | miR-1244 |
| Wang X,2015 | Asian(China) | 59/59 | 55.9/57.6 | 0.70/0.70 | NR | NSCLC(0.53) | I-IV(0.71) | CF | Plasma | miR-486,210 |
| Wang Y,2016 | Asian(China) | 114/134 | 59.8/61.4 | 0.5/0.60 | 0.559/0.478 | NSCLC(1.00) | I(1.00) | HC | Plasma | miR-532,628-3p,425-3p,339-3p |
| Wei J,2011 | Asian(China) | 63/30 | 61/57 | 0.71/0.67 | NR | NSCLC(0.60) | I-IV(0.36) | HC | Plasma | miR-21 |
| Wozniak,2015 | Caucasian(Russia) | 100/100 | 62.6/60.1 | 0.86/0.71 | 0.77/0.64 | NSCLC(0.35) | I-IIIa(0.70) | HC | Plasma | miRNA panel(24) |
| Yan H,2015 | Asian(China) | 300/300 | NR | NR | 0.64/NR | LC((NR) | I-IV(0.34) | HC | PB | miR-31 |
| Yang Y,2015 | Asian(China) | 74/52 | 62.5/61.8 | 0.66/0.65 | NR | NSCLC(0.62) | I-IV(0.54) | HC | PBMCs | miR-10b |
| Yang J,2014 | Asian(China) | 152/300 | NR | 0.64/0.69 | 0.842/0.787 | NSCLC(0.38) | I-IV(0.22) | HC | Serum | miR-152, 148a,148b,21 |
| Yu Y,2017 | Asian(China) | 50/30 | NR | 0.82/0.83 | NR | SCLC(0) | LS, EL | HC | Plasma | miR-92a-2 |
| Zhao W,2015 | Asian(China) | 80/60 | 57.61/55.4 | 0.61/0.52 | NR | NSCLC(NR) | NR | HC | Serum | miR-21 |
| Zaporozhchenko | Caucasian(Russia) | 75/50 | 65/51.2 | 0.89/0.84 | 0.907/0.84 | LC(0.24) | I-IV(0.32) | HC | Plasma | miR-19b,183,25,205,21 |
| Zeng X,2013 | Asian(China) | 64/26 | 60/55 | 0.66/0.58 | NR | NSCLC(0.53) | I-IV(0.53) | HC | PBMCs | miR-143,150 |
| Zhang H,2017 | Asian(China) | 129/83 | 59.3/59.7 | 0.63/0.58 | 0.60/0.46 | NSCLC(0.45) | I-II | HC | Plasma | miR-145,20a,21,223 |
| Zheng D,2011 | Caucasian(USA) | 74/68 | 64.2 | 0.54 | NR | LC(0.24) | I-IV(0.45) | CF | Plasma | miR-155,197,182 |
| Zhou C,2015 | Asian(China) | 91/61 | 58.9/59.4 | 0.73/0.48 | 0.54/0.16 | NSCLC(0.58) | I-IV(0.47) | HC | Serum | miR-652,660,194 |
|  |  | 87/61 | 58.7/56.3 | 0.63/0.52 | 0.51/0.13 | NSCLC(0.60) | I-IV(0.52) | HC | Serum | miR-652,660,194 |
| Zhu W,2014 | Asian(China) | 70/44 | 59/NR | 0.80/NR | NR | NSCLC(0.49) | I-IIIb(>0.51) | HC | Serum | miR-125a-5p,let-7e |
| Zhu W,2016 | Asian(China) | 112/40 | 58.5/57.9 | 0.54/0.55 | 0.357/0 | NSCLC(0.80) | I-IIIb(0.91) | HC | Serum | miR-182,183,210,126 |
| Zhou X,2016 | Asian(China) | 108/94 | NR | 0.43/0.46 | 0.06/NR | NSCLC(1.00) | I-IV(0.52) | HC | Plasma | miR-19b-3p,21-5p,221-3p,409-3p,425-5p,584-5p |
|  |  | 33/30 | NR | 0.46/0.47 | 0.09/NR | NSCLC(1.00) | I-IV(0.52) | HC | Plasma | miR-19b-3p,21-5p,221-3p,409-3p,425-5p,584-5p |
